# Reforming solitary confinement: the development, implementation, and processes of a restrictive housing step down reentry program in Oregon

**DOI:** 10.1186/s40352-021-00151-9

**Published:** 2021-08-26

**Authors:** Ryan M. Labrecque, Jennifer J. Tostlebe, Bert Useem, David C. Pyrooz

**Affiliations:** 1 Department of Criminal Justice, University of Central Florida, 12805 Pegasus Drive, Orlando, FL 32816-1600 USA; 2 Department of Sociology, University of Colorado Boulder, Boulder, CO 80309 USA; 3 Department of Sociology, Purdue University, West Lafayette, IN 47907 USA

**Keywords:** Prison, Administrative segregation, Restrictive housing, Mental and physical health

## Abstract

**Background:**

Over the past decade there have been numerous and impassioned calls to reform the practice of solitary confinement in U.S. prisons. This article examines the development, implementation, and processes of a restrictive housing reentry program in the Oregon Department of Corrections. It draws on data from official documents, site observations, and interviews with 12 prison officials and 38 prisoners. The Step Up Program (SUP) seeks to improve the living conditions in restrictive housing over business-as-usual, alleviate physiological and psychological harms of solitary confinement, and use rehabilitative programming to increase success upon returning to the general prison population or community.

**Results:**

The impetus to change the culture and structure of restrictive housing was primarily the result of internal administrative reform. Prisoners assigned at random to housing assignments offered accounts of their daily activities suggesting that the SUP provides more time out-of-cell and greater access to other services and activities. Program participants preferred the living conditions in the SUP because they had more opportunities for social interaction and incentives for compliant behavior. However, views on the value of programming among respondents were mixed.

**Conclusions:**

The launch of the SUP occurred in early 2020, which was soon followed by the COVID-19 pandemic. As a result, the program was never fully implemented as intended. As Oregon returns to more normal operations, it is possible that the SUP will be able to include even more out-of-cell time, greater socialization opportunities, and increased access to programming and other beneficial activities. As we await the opportunity to conduct prospective psychological and behavioral analyses, this study provides tentative support for the use of step down reentry programs in restrictive housing units.

**Trial registration:**

Open Science Framework, *Preparing adults in custody for successful reentry: An experimental study of a restrictive housing exit program in Oregon*. Registered 4 October 2019, https://osf.io/t6qpx/

*Do we really think it makes sense to lock so many people alone in tiny cells for 23 hours a day, sometimes for months or even years at a time? That is not going to make us safer. That’s not going to make us stronger. And if those individuals are ultimately released, how are they ever going to adapt? It’s not smart.*

– U.S. President Barack Obama, Remarks at the NAACP Conference, July 14, 2015

## Background

Solitary confinement—isolating prisoners for 22 or more hours daily—has become the subject of intense debate and policy reconsideration over the last decade. One controversy focuses on the conditions in restrictive housing settings. While restrictive housing typically does not entail complete isolation or sensory deprivation, meaningful social interaction, and other lifelines (e.g., visitation, phone calls, and programming) are severely curtailed. This has led to extensive litigation based on Eighth Amendment protections against the use of cruel and unusual punishment. For example, the class-action lawsuit *Ashker* v. *Governor of California*, was settled in September of 2015, nearly 6 years after the initial *pro se* complaint was filed (Center for Constitutional Rights, n.d.; Reiter, 2016), ending California’s use of indeterminate placement in restrictive housing.

A second point of controversy has been an alleged overuse of restrictive confinement (Sakoda & Simes, 2021). Around 4% of state and federal prisoners were held in restrictive housing, and nearly 20% spent at least one night in this setting over a 12-month period (Beck, 2015; see also Correctional Leaders Association & the Arthur Liman Center for Public Interest Law, 2020; and Pyrooz & Mitchell, 2020). Restrictive housing is thought to be reserved for the “worst of the worst” (Butler et al., 2013; Reiter, 2012); however, not all individuals held in isolation may need to be separated from the general population to ensure institutional safety and order (Labrecque, 2018; Lovell et al., 2000). Segregation units disproportionately house vulnerable populations (e.g., people with serious mental illnesses; Siennick et al., 2021) and individuals based on their identity rather than behaviors (e.g., gang affiliates; Pyrooz, 2016).

A final point of controversy pertains to the potential for negative effects. Placing prisoners in restrictive housing units is believed by many to exacerbate behavioral and health problems. While the evidence is mixed whether the use of restrictive housing increases the safety of prisons or deters misconduct among prisoners (Labrecque & Smith, 2019a; Morris, 2016; Steiner & Cain, 2016), most studies find some level of psychological or physiological detriment (Haney, 2020; Luigi et al., 2020; Morgan et al., 2016; Reiter et al., 2020; Strong et al., 2020; Wildeman & Andersen, 2020).

Just as there has been a swing away from the excesses of mass incarceration after decades of unbridled growth (Lobuglio & Piehl, 2015), a new consensus has emerged from these controversies that it would be desirable to reduce the scale and scope of restrictive housing in U.S. prisons (see Garcia, 2016).

### Strategies to reduce the use of restrictive housing

Broadly speaking, there are three types of approaches to achieve the goal of shrinking the footprint of restrictive housing. One approach has been to address the “on-ramps”—tightening the guidelines for whom or what qualifies for placement in restrictive housing settings. Prisoners are typically placed in restrictive housing for disciplinary reasons, such as rule violations, or administrative reasons, such as membership in security threat groups (Labrecque, 2016; Mears, 2016). Abolitionists seek to eliminate restrictive housing altogether, while defenders of the practice contend that some level of its use is required to respond to the challenges of disorder and violence in prison (see Labrecque & Mears, 2019; Mears & Castro, 2006). In 2016, the Federal Bureau of Prisons reduced the maximum punishments for disciplinary sanctions and eliminated juveniles from being placed in restrictive housing (Office of the Press Secretary, 2016). Another on-ramp strategy involves proactively targeting those who are at higher risk for ending up in restrictive housing with increased case management and treatment services in order to reduce the need for placement (Labrecque & Smith, 2019b).

A second approach is to address the “off-ramps”—reducing the dosage of restrictive housing. Shortening the length of stay in these conditions would require eliminating the practice of indeterminate placement. Gang affiliates, for example, would no longer have to debrief in order to return to the general prison population (Toch, 2007). It would also require limiting fixed sentences to restrictive housing. Approximately 10% of federal and state prisoners reported spending 30 or more days in restrictive housing in the last 12 months (Beck, 2015). That proportion would drop even further, allowing U.S. prisons to fall more in line with the United Nations’ 2015 standards of prisoner treatment, which prohibits placement in restrictive housing for 15 days or more (Frost & Monteiro, 2016). Among the 50 guiding principles set out by the U.S. Department of Justice (2016) is that correctional agencies should develop plans that result in the prompt return of prisoners to the general population.

A third approach—and the subject of this article—involves altering the environment within restrictive housing settings. Whereas the theory of change for the prior approaches entails restricting the on-ramps and expanding the off-ramps, this approach targets the mechanisms believed to bring out problem behavior and poor health among the people who are placed in restrictive housing, namely the excessive deprivations and limited social interaction with others (see Haney, 2020). First, one of the most frequently mentioned harms of solitary confinement by criminologists, psychologists, and public health experts alike is the lack of *meaningful* social contact experienced in these units (Cloud et al., 2015; Haney, 2020; Scharff-Smith, 2006). This is because restrictive housing units provide limited to no opportunities for time out-of-cell or social interaction (e.g., phone calls, visitation, hanging out with other prisoners, engaging with correctional staff). Prison culture is also inherently coercive in nature (Wooldredge, 2020), which can impede the positive interactions between prisoners and correctional staff and increase non-compliance and other forms of rule breaking (e.g., Beijersbergen et al., 2015; Steiner & Wooldredge, 2018). Providing more opportunities for time out-of-cell, quality social interaction, and cultural changes away from coercion should lessen incidence of misconduct and improve indicators of health and psychological well-being. Second, and relatedly, there is a lack of rehabilitative programming geared toward reintegration and increasing pro-social behavior through problem solving and conflict resolution (Butler et al., 2018; Meyers et al., 2018, 2020). As many individuals are placed in restrictive housing settings because of rule violations and threat assessments, by restricting access to programming, it is unlikely these behaviors or the threat will be reduced. Finally, there are minimal incentives for compliance in restrictive housing units. Incentives such as extra phone calls, opportunities to play card and video games with others on the unit, and access to a wider food select can be powerful tools to encourage compliance and boost morale (Digard et al., 2018; Smith, 2016).

This approach aims to construct an environment that permits people to leave restrictive housing in a better position than when they arrived. Elements of a “better” environment include: (1) allowing more time out-of-cell, higher quality interaction between prisoners and staff, and more amenities, (2) incorporating rehabilitative programming into restrictive housing units with the goal of reintegrative success, and (3) providing incentives for compliant behavior that are comparable to the privileges found in general population housing. If implemented, these changes are expected to improve prisoner well-being (including physical and psychological health), decrease problem behavior, and reduce their likelihood of being sent back to restrictive housing after returning to the general prison population.

Across the United States, prison systems have developed what has been termed “step down programs” in restrictive housing settings. Their purpose is to help prisoners transition from more to less restrictive conditions (Ghafar, 2017; Vanko, 2019). These programs contain two or more levels that provide prisoners with increasingly greater privileges with each “step.” They may gain more recreational time, access to the canteen, contact with family, or opportunities to engage in rehabilitative programming.

### The current study

It is estimated that as many as 30 prison systems operate a step down program (Association of State Correctional Administrators & the Liman Center for Public Interest Law, 2018; Chammah, 2016); however, information on the origins, implementation, processes, and impacts of these programs is largely absent from the social science literature. Toward the goal of filling key aspects of this research gap, we focus on the task set forth by the Oregon Department of Corrections (DOC) to create a new unit that is committed to rehabilitative programming, increased socialization opportunities, incentivizing reintegration, and blunting some of the harsher conditions of restrictive housing.

This article examines the development and implementation of a restrictive housing step down reentry program in an Oregon prison. It is organized into three sections. First, we outline the research methodology of the evaluation generally and for this article specifically. Our data are based on official documentation, observations, and interviews with prison officials and prisoners with respect to two housing units: (1) the newly-devised Step Up Program (SUP) unit, and (2) the business-as-usual Intensive Management Unit (IMU). Second, we describe the origins of the SUP, including what motivated its development, the procedures for putting it into place, and opinions about it from the perspective of correctional staff tasked with its implementation. Finally, based on interview data with prisoners we describe their use of time and perceptions of programming, comparing prisoners randomized to placement in the SUP and those who remained in the IMU. These results are not intended to serve as a complete evaluation of the program, but instead to share knowledge on an operational innovation in light of the coronavirus (COVID-19) pandemic’s far-ranging effects on prisons (Novisky et al., 2020). In response to the COVID-19 pandemic, the Oregon DOC prevented outside visitors from entering its prisons and reduced programs and other services and activities, including temporarily suspending the SUP referral process in April 2020.^1^

## Methods

The authors coordinated with correctional staff to conduct a randomized controlled trial (RCT) of the SUP. This is noteworthy for two reasons. First, researchers are typically involved in post-hoc evaluations of policies, practices, and programs. In this study, however, we were able to observe the origins and development of the SUP, which permitted our team to produce a richer account of processes and impacts. It also allowed us to share knowledge on practical and theoretical advances derived from research on restrictive housing, helping the DOC make decisions about the contours and functions of the SUP. Second, RCTs are rare in prisons (Farrington & Welsh, 2005; Weisburd, 2000) and within restrictive housing settings more specifically (Butler et al., 2018; Meyers et al., 2018, 2020). The use of an RCT in this context is important because this strategy reduces the problem of selection bias (Elwert & Winship, 2014; Ridgeway, 2019), which is common in research on restrictive housing.

### Study design and procedure

This study assesses prisoner viewpoints of the living conditions in the SUP and IMU, as well as the prison officials’ perceptions of the SUP as it was being implemented. This research was approved by the Oregon DOC Office of Research and by the Institutional Review Boards at the authors’ respective institutions. A non-security staff member from the Oregon DOC compiled a list of prisoners as they were admitted into the IMU at the Snake River Correctional Institution (SRCI).^2^ The IMU security staff then reviewed the list of names with input from the correctional officers in the unit to determine eligibility for the SUP. Reasons for exclusion included recent attacks against staff, history of violence with a weapon, or otherwise posing an immediate and serious threat to safety and security. Additionally, if a prisoner was scheduled to be released directly to the community from IMU they would automatically be placed in the SUP.^3^ This, of course, limits the inferences that can be made about the impact of SUP versus IMU on outcome variables. However, this paper is centered on description and process, not the impact of the program.

The list of eligible cases was de-identified and sent to the first author for random assignment into either the treatment group (i.e., SUP) or the control group (i.e., IMU). RCTs may raise ethical concerns in the correctional context because they deliberately withhold a “treatment” from some individuals to allow for an unbiased comparison with those who are untreated. In this study, random assignment was reasonable because bed space in the SUP was limited. Oregon officials needed a strategy to assign prisoners to the SUP who met the eligibility criteria and random assignment provided a fair procedure. This strategy also provided the opportunity for the collection of high-quality evidence to evaluate the program’s effectiveness. Still, even though prisoners understood that the invitation to enter the SUP or remain in the IMU was random, they were not mandated to move, and in fact, many exercised their right to remain in the IMU (see below). Declination to move to the SUP had no impact on prisoners’ incarceration, generally, or duration spent in the IMU, specifically.

The first cohort of randomized study participants was moved to the SUP on January 7, 2020. For those assigned to SUP, the average length of stay in IMU before the transfer was 33 days (*SD* = 17 days). The minimum number of days was 11 and the maximum was 72. Thus, while all prisoners had some exposure to IMU, that exposure for the SUP participants was much shorter than the 7 months (~ 213 days) individuals placed in IMU normally experience in this setting.

Our research strategy involved interviews with prisoners. These interviews occurred about 2 weeks prior to their release from SUP or IMU. Travel to SRCI for prisoner interviews was scheduled to occur April 2020. This trip, however, was cancelled when SRCI was closed to outside visitors to slow the spread of the COVID-19 virus (Oregon Department of Corrections, 2021a). Despite this logistical setback, our research team collaborated with prison officials to devise an alternative strategy for conducting the interviews by phone (Pyrooz et al., 2020). We interviewed prisoners in private attorney rooms located within the IMU. Correctional staff first notified prisoners of the study at their cell, asking if they wanted to speak with us voluntarily; some refused, and thus did not participate in the study. If they agreed to learn more about the study, prisoners were escorted to the location where non-security staff dialed our phone number. These interactions were assured the privacy protections of an attorney call; staff did not monitor or record the conversations. We initiated the phone call with our consent form. After obtaining verbal consent, we administered the survey instrument and audio-recorded the interview with the prisoner’s permission. Information about our response and participation rates is provided below.

The SUP stopped accepting new participants in April of 2020 due to COVID-19 but continued to operate in a modified format for those already enrolled through August. In an effort to slow the spread of the virus, congregate recreation and program activities were eliminated. The program staff, nevertheless, endeavored to safely get SUP prisoners out of their cells as much as possible. This involved single-man outdoor recreation time and the incorporation of packet-based programming that could be completed alone in one’s cell. Program participants were afforded greater access to the phone and other benefits, such as more access to television, canteen privileges, and out-of-cell time, compared to those in the IMU.

### Participants

The authors conducted semi-structured interviews with 12 prison officials at the SRCI in private rooms. A purposive sampling method was used to identify staff of different ranks and duties, including correctional officers, case managers, and mid-level and senior administrators. The goal of these interviews was to ascertain staff views on the IMU and SUP, including the motivation and processes behind the development of the SUP. The interviews ranged from 20 min to just over 1 h in length.

A total of 56 prisoners from seven cohorts were enrolled in the study between January 7 and April 21, 2020, of whom 30 were randomly assigned to the SUP treatment condition and 26 were randomly assigned to the IMU control condition. Thirteen of the individuals refused to leave their cell to learn more about the study and three declined to participate at the consent stage (71.4% response rate, 93.0% participation rate). Two respondents provided consent but completed only a partial interview, missing the key sections reported in this study. The 38 consenting participants were evenly split between the treatment and control group. The authors conducted these structured interviews remotely on four multi-day occasions between April 3 and June 30, 2020.

Table 1 contains descriptive statistics based on administrative data for the full sample (i.e., all people randomly assigned) and the analysis sub-sample (i.e., those who were interviewed) partitioned by their random assignment status. Random assignment produced a well-balanced distribution of SUP participants and IMU control group members. In the analysis sample, there were two individuals assigned to the treatment condition who refused to participate in the SUP and three individuals assigned to the treatment condition who began but were terminated early from the SUP for violating institutional rules.

**Table 1:** Demographic and Institutional Descriptive Statistics, Total and Analysis Samples

	Total Sample					Analysis Sample				
Characteristic	% Total (*N* = 56)	% IMU (*n* = 26)	% SUP (*n* = 30)	*t* or *z* score	*p*-value	% Total (*N* = 38)	% IMU (*n* = 19)	% SUP (*n* = 19)	*t* or *z* score	*p*-value
Mean age (SD)	29.8 (6.9)	29.0 (6.4)	30.6 (7.2)	0.89	.377	30.1 (6.7)	29.5 (7.0)	30.7 (6.6)	0.55	.587
Race/ethnicity										
*White*	57.1	50.0	63.3	1.01	.315	52.6	57.9	47.4	−0.65	.516
*Black*	8.9	11.5	6.7	−0.64	.524	10.5	10.5	10.5	0.00	1.000
*Hispanic*	26.8	23.1	30.0	0.58	.560	29.0	15.8	42.1	1.79	.074
*Asian*	1.8	3.9	0.0	−1.08	.278	2.6	5.3	0.0	−1.01	.311
*American Indian*	5.4	11.5	0.0	−1.91	.056	5.3	10.5	0.0	−1.45	.146
Gang affiliation	76.8	73.1	80.0	0.61	.541	76.3	68.4	84.2	1.15	.252
Mental health										
*MH-0*	28.6	23.1	33.3	0.85	.397	26.3	26.3	26.3	0.00	1.000
*MH-1*	28.6	19.2	36.7	1.44	.150	23.7	10.5	36.8	1.91	.056
*MH-R*	30.4	53.9	10.0	−3.56	<.001	36.8	63.2	10.5	−3.36	<.001
*MH-2*	12.5	3.9	20.0	1.82	.068	13.2	0.0	26.3	2.40	.016
ACRS										
*Low-risk*	48.2	42.3	53.3	0.82	.410	52.6	42.1	63.2	1.30	.194
*Moderate-risk*	50.0	53.9	46.7	−0.54	.592	44.7	52.6	36.8	−0.98	.328
*High-risk*	1.8	3.9	0.0	−1.08	.278	2.6	5.3	0.0	−1.01	.311
Prior IMU	44.6	38.5	50.0	0.87	.386	44.7	36.8	52.6	0.98	.328

*Note:* IMU = Intensive Management Unit; SUP = Step Up Program; MH = Mental Health; ACRS = Automated Criminal Risk Score. *p*-value refers to IMU to SUP differences derived from two-tailed t-tests or equality of proportions tests

The respondents in our analysis sample were between 20 and 45 years old at the time of the interview (*Mean* = 30.1, *SD* = 6.7). The sample was predominately White (52.6%), followed by Hispanic (29.0%), Black (10.5%), American Indian (5.3%), and Asian (2.6%). Approximately three-quarters of the sample (76.3%) were identified as having a history of gang affiliation and nearly half (44.7%) had been housed in the IMU on a prior occasion. According to the Automated Criminal Risk Score (ACRS) instrument, 52.6% of the respondents were classified as low-risk, 44.7% as moderate-risk, and 2.6% as high-risk for post-release recidivism (i.e., a felony reconviction within 3 years of release).

With regard to mental health, the agency classifies prisoners into five categories, ranging from less severe to more severe: MH-0: does not require a diagnosis by mental health services (26.3% of respondents); MH-1: diagnosis of mental disorder with mild acuity (23.7%); MH-R: diagnosis of a condition that either needs no active therapeutic intervention or symptoms that can be controlled through medication (36.8%); MH-2: diagnosis with one or more of 19 mental disorders, including anxiety, depression, mood, and panic disorder (13.2%); and MH-3 diagnosis with one or more of nine severe mental disorders, including bipolar, schizophrenia, and psychotic disorder (0.0%).^4^

The two groups (IMU vs. SUP) were statistically similar (*p* > .05) with two exceptions. In the full sample (*N* = 56), a lower percentage of the SUP group maintained an MH-R classification than the IMU group (10.0% compared to 53.9%, *p* < .001). In the analysis sample (*n* = 38), the SUP group had fewer MH-R (10.5% vs. 63.2%, *p* < .001) and more MH-2 designations than the IMU group (26.3% vs. 0.0%, *p* = .016). With 15 comparisons, this is about what we would expect to occur by chance. With the exceptions noted, there were no statistically significant differences found on any of the observed characteristics between the SUP analysis and total samples or IMU analysis and total samples. Randomization resulted in well-balanced groups.

### Prisoner survey

Components of the interviews with prisoners focused on time use in prison and perceptions of the SUP. *Time use* questions were based on the Survey of Inmates in State Correctional Facilities (SISCF-2004) and involved asking respondents to describe their typical day in custody, estimating how much time they spent engaged in various activities (e.g., participating in yard time, hanging around with other prisoners, participating in programming, communicating with correctional officers). *Perceptions of the SUP* involved inquiry about prisoners’ views of the program, including asking them to detail how the experience differed from the IMU and to comment on expectations regarding its potential impact on their future.

### Analysis

This study employs a mixture of descriptive, quantitative, and qualitative analyses. First, we examine the origins of reforming the IMU and the development and implementation of the SUP, relying on historical accounts, observations of the unit, and interviews with prison officials. Second, we compare the daily reported activities between participants in the SUP and the IMU to determine if there are differences in the experiences of prisoners in the respective housing units. Consistent with an intent-to-treat approach, we make comparisons based on the assigned treatment condition (Lachin, 2000),^5^ but given the descriptive aims of the study, we also make comparisons based on actual housing assignment at the time of the interview. Finally, we review the transcribed responses to the open-ended questions about the perceptions of the SUP and IMU.^6^ This study takes an inductive approach to the analysis of the qualitative data. Each of the authors read the transcripts to identify and collate common themes and conclusions.

We are cautious in the interpretation of our quantitative findings. Indeed, we focus on three aspects of differences between groups, including the direction (i.e., positive or negative), statistical significance (i.e., alpha values), and magnitude (i.e., strength of the association). Statistical significance is determined through the use of independent samples *t*-tests, and we report *p-*values albeit without asterisks. Substantive significance is assessed through the interpretation of Cohen’s *d*. We follow Cohen’s (1988) guidelines where *d* = .2 represents a small difference, *d* = .5 represents a medium difference, and *d* = .8 represents a large difference in the magnitude of the SUP effects, using the IMU respondents as the reference group. Using multiple modes of assessing differences is important for a study design involving an analytic sample of this size.

## Results

### Origins of reform

The origins for reforming the structure and culture of restrictive housing in Oregon are found in two events. First, in 2015, the Oregon DOC applied for and was selected as one of five correctional agencies to participate in the Vera Institute of Justice Safe Alternative to Segregation Initiative (Digard et al., 2018). This project involved the review of Oregon’s restrictive housing policies, observation of correctional facilities, focus groups with staff and prisoners, and administrative analysis of segregation data. Vera found that prisoners were often being isolated in segregation cells for up to 23 h per day with limited opportunities for out-of-cell activities and therapeutic programs. Vera recommended that the Oregon DOC should seek to improve the conditions of confinement in restrictive housing settings (e.g., create de-escalation spaces), increase the availability of out-of-cell programming and congregate activities, and create a structured reentry process to help prisoners transition more effectively out of long-term segregation (see also Hastings et al., 2016). According to an Oregon DOC administrator that we spoke with:

Vera made the recommendation [for the step down program]. They liked the fact that we had started the out-of-cell programming, but they recommended, of course, that we do more and that we work on a step down program that [would] get folks ready for a general population.

Thus, Vera provided an impetus to reform the structure of restrictive housing in Oregon.

Second, the Oregon DOC was also chosen to take part in a correctional staff exchange program with the Norwegian Correctional Service in 2018 (Achen, 2018).^7^ A delegation of 14 Oregon correctional administrators and officers traveled to Norway in 2019 for 9 days to observe their prison operations and to stay in the homes of their Norwegian counterparts, and vice-versa (Frost, 2019). The Norwegian correctional system is often touted for its efforts to make prisons more “humane” and normalize prisoner/officer interactions. Indeed, a senior administrator stated: “ … that’s what Norway is about. They say it’s the recognition that we’re all people who still have hopes and dreams.” The impetus behind the exchange program was a belief that correctional staff would better support and implement more humanistic and less punitive offender management strategies after observing first-hand the benefits of this strategy in practice (Ahalt et al., 2020). It was expressed to us that the benefits of the Norwegian approach were readily apparent during the exchange trip:

I think what we really saw were two things that were really kind of stunning to us … one [is] just their rapport and relationships with inmates … Everything was done together. There was none of this “us” and “them.” They had professional boundaries, and they were appropriate, but there was much more integration, so the whole atmosphere and the tone were different … The other takeaway that was really big was just how happy and healthy this staff seemed to be … So, we saw that how they had their system not only benefited the inmates and helped them get ready for release, they have the lowest recidivism in the world, but their staff are benefiting from it as well.

As part of the exchange program, correctional staff from the Norwegian Correctional Service were reciprocally sent to the Oregon DOC:

They … train [ed] us on … their concept of the resource team that they use with their difficult inmates in restrictive housing populations. I think some of the easy things for Americans to believe about Norway is they can do all that because they have easy inmates. They don’t have easy inmates. Their incarcerations and sentencing are different than ours, but they have rapists, murderers, and terrorists just like we do, so some of the things they’ve done with their resource team are really profound. We were trained on that in February of 2019, and that has really been a real catalyst of support in some of the culture change in things we’re doing in our step up program.

The exchange thus contributed to alternative ways of thinking about the culture found in restrictive housing.

These two events, coupled with the agency Director’s goal of reducing the use of restrictive housing (Oregon Department of Corrections, 2021b), have corresponded with a 30% statewide reduction in the use of the practice. Out of the 14,734 prisoners incarcerated in July 2019, there were 705 (or 4.8%) who were held in some type of restrictive housing (Correctional Leaders Association & the Arthur Liman Center for Public Interest Law, 2020), which was down from the 1025 out of 14,591 (or 7.0%) from Fall of 2014 (Baumgartel et al., 2015).

Building from this momentum, the Oregon DOC sought to implement a restrictive housing step down reentry program with the objectives of providing a transition that would help prisoners more effectively move out of long-term segregation and improving prisoner-correctional staff interactions by putting the “person” back in the “prisoner.” The Oregon DOC selected the IMU at Snake River Correctional Institution (SRCI) in Ontario (located in eastern Oregon near the Idaho border) as the site for its first location.^8^

### Creating an alternative to business-as-usual

The IMU is reserved for individuals who are believed to pose a serious risk to the safety of themselves, others, or the institution (e.g., chronic rule violations, escape attempts, security threat group activity).^9^ All referrals to IMU are reviewed by a multi-disciplinary committee, which includes representatives from institutional operations, behavioral health services, and the office of population management. Placement in IMU involves confinement in a single cell for the vast majority of each day (~ 23 h per day) with little-to-no access to meaningful interactions with staff or other prisoners. Stays in IMU are slated to last 7 months with compliant behavior; however, time in the unit can be extended for violating institutional rules. Individuals with serious mental illnesses can alternatively be placed in the Behavioral Health Unit, where they are more closely monitored by medical and mental health providers.

The IMU operates on a five-level tiered system—Level 1 to Level 5—with prisoners assigned to lower levels having more restrictions on services and activities than those in higher levels (e.g., property, commissary, recreation, phone, visits, work; Oregon Department of Corrections, 2019). All prisoners enter IMU under a Level 2 program status and receive a behavior action plan from their assigned case counselor. The behavior plan outlines the specific programs and other activities that the prisoner must complete before being considered for release from IMU. Each month the multi-disciplinary team meets to review the program status for all of the prisoners in IMU. During these meetings, the committee decides to advance, demote, or retain prisoners’ assigned program level.

The SUP was designed with the goals of improving living conditions compared to the IMU, alleviating potential physiological and psychological harms of restrictive housing, and increasing one’s success upon returning to the general prison population or community. As part of the planning process for this transition, the authors provided guidance on the structure of the unit based on the current inventory of literature. For example, it was recommended that the new unit provide more out-of-cell time, increased social interaction, and more opportunities for rehabilitative treatment compared to IMU. The design of the program (e.g., types of programs to be included, phase/incentive system, expanded recreation yard, video game system in unit), however, was decided exclusively by staff at the Oregon DOC. The SUP was developed to include two levels. Prisoners in Phase 1 can take part in out-of-cell activities at tables in the common area while restrained and outdoor buddy recreation with one other person in the unit. Those in Phase 2 have access to out-of-cell activities while partially restrained (i.e., legs cuffed to table, but arms are free), buddy recreation with up to three other people in the outside recreation area, and unrestrained escorts. The authors also collaborated with Oregon DOC senior managers, SRCI administrators, and IMU security, program, and support staff to devise a strategy for evaluating the impact of the program on a variety of physiological, psychological, and behavioral indicators.

The process for converting a wing of the IMU at SRCI into a 24-person, step down reentry program involved five critical tasks. First, the new unit required physical modifications to allow for greater socialization and out-of-cell time compared to the IMU. The outdoor recreational area was expanded to provide more space for prisoners to congregate together. Tables were also installed in the unit to allow prisoners the opportunity to sit and talk, play games together, and watch television outside of their cells.

Second, opportunities for social interaction were significantly increased. A goal was set to provide program participants with 5–6 h of out-of-cell time per day, which is more than the 1 h per day that prisoners in the IMU receive.^10^

Third, given the desire to improve the likelihood for a successful reentry, treatment services and other activities were enhanced in the SUP. This involved increasing the number of programs, including Free Your Mind in Segregation (FYM-S); Dialectical Behavioral Therapy (DBT); Getting Out by Going In (GOGI); Living Well; Hepatitis, HIV, AIDS Awareness Program (HHAAP); and Charting a New Course. It also involved providing opportunities for other recreational activities, such as watching movies, playing games, and participating in art projects, that are not available to those in the IMU.

Fourth, a correctional case counselor was assigned to oversee the treatment plans of all the program participants. The counselor assigns prisoners to the specific programs and other services available that best match their individual risk and needs. Although prisoners in both SUP and IMU work with a case counselor, those in the former category have much greater access to programs and services that address their risks and needs.

Finally, following the Norwegian model, correctional officers have been encouraged by SRCI leadership to take a more humanistic approach toward interacting with prisoners in the unit. This approach includes spending time with incarcerated individuals on the unit by playing cards, video games, or other activities together, which are not typical occurrences in the IMU. According to a senior security staff member:

To change a culture, you’ve got to drive the culture and show people it’s okay. It’s okay to go sit down at the table and play video games with inmates. It’s okay to go sit down and observe cards or play cards … You’ve got to role model that … it’s okay to talk to somebody about their family if they ask you. I do care about your family. I do care about your kids. I do care about their success. I know you’re an inmate, but that’s important to you, so it needs to be important to me. [L] ater that’s going to pay dividends. I promise you. One case at a time.

### Implementing the step “up” program

The SUP was piloted on October 21, 2019. Its first residents included eight hand-selected prisoners from the IMU. Three additional individuals were also identified for placement but refused to participate. Correctional staff informed us that the trepidation toward program participation had to do with gang politics, unknowns about the program, and the original program name itself—“Step *Down* Program.” Our research team traveled to SRCI in late October 2019 for a kick-off meeting with more than two dozen line to senior staff members. During this meeting, we summarized the state of knowledge on restrictive housing and offender rehabilitation. We also discussed the motivation for the project and the research evaluation plan. During this meeting, an IMU correctional officer asked about changing the name of the Step Down Program because it was signifying to some prisoners that it was a gang drop-out program. A suggestion was made to instead call it the Step Up Program (SUP), which had a more positive connotation. After a unanimous vote among staff at the meeting, the name of the program was officially changed.

During our site visit, we toured the IMU and saw the modifications to the SUP. We also interviewed a range of correctional officials and staff about their perspectives on the new program. When asked about the goals of the SUP, one of the staff members commented, “I think that the most important thing is to get these guys out of the cell and communicate with each other and socialize.” Other staff members reported a general optimism regarding the potential benefits of the program on participants, other prisoners, staff, and the institution.

In my opinion, [the SUP is] really to make them successful when they do move out into that [general population] setting. It doesn’t take long for somebody to get uncomfortable with the [general population] setting when they’ve been in [restrictive housing]. We’ve got some of these guys that have been in there a long time. There’s no way I can feel comfortable saying, “Yeah, let’s take this person whose been in this setting and just throw them back in to this big pond and see what happens.” If we want them to be successful then we need to gradually work them out to that … They’re either going to panic and do something, just enough, to get them back, or they’re going to panic and freak out and do something major that’s going to hurt somebody.

In the view of another staff member:

Anytime we can get a guy out of restrictive housing with a lower anxiety level, that’s going to make it safer to the officer supervising the unit, the other [prisoners] in the unit. They’re not going to be on edge because they’re already used to those interactions. So basically, we are taking advantage of having the ability to test and observe in a controlled environment before we just take the cuffs off and say, “All right. Go be successful.”

However, there was also some trepidation expressed about the program voiced among correctional staff:

As with all change I think people are really apprehensive of things, especially with dealing with people because they’re unpredictable. But I would say that a majority, yeah are supportive of it, especially the staff that worked down here just because we’re so informed … We’re more informed because we’re down here [on the unit], but the [general population] staff, maybe not just because they’re not informed.

Prisons are typically a beehive of activities, replete with routines and roles that are stable. Change can be difficult, even if it is perceived as being for the better. One staff member believed that others would come around to the SUP:

Some of the things that, earlier on, that folks were negative about, they’ve quickly learned that there’s things that work. Some of them even admit, they were so negative about something and then once they actually saw it implemented and they saw the results of it, it was like wow … The staff that have witnessed that themselves or have been part of it even when they were begrudgingly part of it, we’ve seen a lot of attitude change. You’re always going to have the little pockets of negative. That’s fine.

In addition to the tour and staff interviews, we also pilot tested our survey instrument with prisoners from the first cohort of SUP participants, drawing on this experience to identify ways to refine and improve our survey tool.

### The SUP in practice

To assess if the living conditions in SUP were modified as intended, we asked respondents how much time they spent in the last week engaged in a variety of activities. Table 2 compares the findings of the study participants assigned to the SUP and IMU as well as those housed in the SUP and IMU at the time of the interview. The average daily amount of *time spent out of one’s cell* was much greater for the participants assigned to the SUP compared to those who were assigned to the IMU (112.9 min compared to 47.4 min, *d* = .91, *p* = .008). Although not statistically significant at the .05 level, there was a moderate positive association detected between treatment group assignment and two activities: *hanging around with other prisoners* (*d* = .60) and *watching TV* (*d* = .55). There was also a smaller positive association between treatment assignment and *participating in programming* (*d* = .44), *engaging in physical exercise* (*d* = .35), *writing* (*d* = .35), and *participating in yard time* (*d* = .23), and a small negative association found for *reading* (*d* = −.47) and *communicating with correctional officers* (*d* = −.27). There were no substantive differences found between the two groups on the three activities of *drawing*, *talking on the phone*, or *engaging in hygiene activities* (i.e., absolute values of Cohen’s *d* < .20). To summarize, we are observing meaningful differences in the living conditions between SUP and IMU housing.

**Table 2:** Group Differences in Average Daily Time Use (in Minutes) Over the Last Week, Intent-to-Treat and Housing-at-Interview Samples

	Analysis Sample		Intent-to-Treat Sample						Housing-at-Interview Sample					
	(*N* = 38)		IMU (*n* = 19)		SUP (*n* = 19)			*p*-value	IMU (*n* = 24)		SUP (*n* = 14)			*p-*value
	*Mean*	*(SD)*	*Mean*	*(SD)*	*Mean*	*(SD)*	*d*		*Mean*	*(SD)*	*Mean*	*(SD)*	*d*	
Time out of your cell	80.1	(75.6)	47.4	(68.2)	112.9	(75.6)	+ 0.91	.008	44.4	(61.7)	141.4	(66.0)	+ 1.53	.000
Participating in yard time	33.8	(33.5)	29.9	(41.6)	37.7	(23.3)	+ 0.23	.478	30.5	(38.2)	39.4	(23.5)	+ 0.26	.438
Watching TV or on a tablet	234.6	(149.7)	194.5	(143.8)	274.7	(148.4)	+ 0.55	.099	209.0	(137.8)	278.6	(164.1)	+ 0.47	.170
Reading books, magazines, or other non-religious material	169.1	(131.7)	199.9	(145.6)	138.3	(111.7)	−0.47	.152	173.9	(139.5)	160.9	(121.8)	−0.10	.774
Hanging around with other prisoners	85.4	(175.7)	33.9	(89.7)	136.8	(223.3)	+ 0.60	.071	34.4	(81.0)	172.8	(251.5)	+ 0.84	.017
Doing physical exercise	74.2	(43.1)	66.6	(35.2)	81.9	(49.6)	+ 0.35	.282	65.2	(32.2)	89.7	(55.2)	+ 0.58	.092
Writing	86.9	(90.5)	71.2	(96.5)	102.6	(83.7)	+ 0.35	.291	77.6	(89.7)	102.7	(93.0)	+ 0.28	.416
Drawing/art	43.7	(72.9)	38.7	(66.5)	48.7	(80.2)	+ 0.14	.678	44.4	(73.1)	42.5	(75.2)	−0.03	.940
Participating in programs, education, counseling	17.1	(27.6)	11.1	(22.8)	23.1	(31.0)	+ 0.44	.181	11.3	(21.3)	27.1	(34.4)	+ 0.59	.088
Communicating/hanging out w/ correctional officers	3.2	(6.6)	4.1	(7.5)	2.4	(5.6)	−0.27	.414	3.3	(6.8)	3.1	(6.4)	− 0.03	.925
Showering, brushing your teeth, etc.	39.6	(33.0)	38.2	(28.9)	41.1	(37.4)	+ 0.08	.796	37.3	(26.5)	43.6	(42.7)	+ 0.19	.582
Talking on the phone with friends and family	8.3	(11.2)	8.9	(12.7)	7.7	(9.7)	−0.10	.753	8.9	(12.1)	7.3	(9.8)	− 0.14	.685

*Note:* IMU = Intensive Management Unit; SUP = Step Up Program. *d* refers to standardized differences between individuals in the IMU (control group; m_1_) and those in the SUP (experimental group; m_2_). *p*-value refers to IMU to SUP differences derived from two-tailed t-tests

Table 2 also compares the responses of individuals based on their housing location at the time of interview, as five of the 18 people randomly assigned to the SUP either did not enter the SUP (i.e., declined) or prematurely left the SUP (i.e., removed due to misconduct). The results are substantively similar but less noisy. For example, the number of minutes out-of-cell increased to 141 from 113, while the standard deviation shrunk to 66 from 76 min. The time spent hanging out with other prisoners also increased to 172 from 137 min. These are positive signs that, despite the pandemic, the SUP was operating as intended, lessening the deprivations of restrictive housing.

The qualitative data also suggested that the living conditions in the SUP were preferable to the IMU. When asked to describe the biggest difference between the two units, SUP participants reported having considerably more opportunities for meaningful social interaction with other prisoners. One respondent stated:

Well just being in close contact with other prisoners, that’s the biggest difference … [I] n regular IMU, the only time you talk to somebody is through the crack of your door. You’re not talking to other prisoner’s face to face … So just that contact around other people is a big difference.

Others saw value in the additional incentives that were available in the unit. One individual noted:

You get two more phone calls a week. Once you get your level three, you get two more phone calls. We go to yard with somebody, like we know somebody on the unit with us, we can go to yard with them. It just breaks up your time better … Well it’s just more privileges … Once you get to phase two, you’re not cuffed up no more to be moved around from in the unit to come out to yard or to go to the day room. We get day room time, so we get to come out and play video games or board games, stuff like that. We go outside to rec with another person.

The benefits of the SUP were not limited to out-of-cell time and social interaction. Another respondent commented:

We can buy more stuff on the canteen. There are just little things that you don’t think about while you’re on mainline [general population], but when you come to IMU, you miss out on.

The program participants, however, shared mixed views on the value of the programming available in the unit. When asked if the amount of time spent in the SUP was enough to make a difference in their future, some responded with a positive view, such as:

Yes, I do.. . It helps me interact with people to where I’ll be able to be already somewhat used to it, as far as just going straight out to mainline [general population] and being not able to be around people.

Another prisoner responded:

I’ll be honest, I’m kind of a knucklehead. The classes that are in this program... helped me sit myself down and really evaluate my life, and it’s given me tools... to deal with calming yourself down or what you’re going to do in a heated moment, real quick if there’s a way to get around certain situations that might land you in trouble, and it’s helped me evaluate more things and brought me closer with my family.

He added that programming provides opportunities for interaction with other individuals:

Another thing that I feel helps is you get to come out of your cell twice a day. You can go out to yard with one of your friends, and then you can go work out. And then you can get to come out and play PlayStation 4 or play cards at a table with people that you’re compatible with. So, you get that interaction that you would kind of like being on mainline. It makes the time go by really easy.

Others, however, saw little value in the programming in the SUP as currently implemented. As one prisoner put it:

Okay. If a person truly wanted to change … if they truly wanted to change and this program was supposed to help make that a success, then yeah, they would need more time in it. I don’t really see what the goal is as of yet … yeah there would need more of it, it’s not sufficient.

Another prisoner responded:

I think we only have one [program] being in the Step Up Program, which is... I don’t even know the name of it, I can’t think of it, but it’s only like 30 min. You know what I mean? When most classes are about an hour, hour and a half.

One individual also discussed the negative impact of COVID-19 on the opportunities for socialization and treatment services in the SUP:

What I think it all boils down to is coming back to social interaction and stuff like that and being in a setting where you would be if you were placed in civilization and society and working from those angles, when they basically throw a book in your cell and you’re just sitting there doing book work. You gain stuff if you’re really paying attention but I mean, who does that? Nobody does that in the real world. You don’t just isolate in a box and write shit down in a notebook. That’s crazy. ... And mind you we’re going through that Coronavirus, so I will say that there is a couple options that were presented that were in a classroom setting that are not applicable at the moment, because they’re trying to not let that spread or whatever, I guess.

To summarize, prisoners viewed SUP as providing them several advantages over the standard restrictive housing setting (i.e., IMU). The SUP configuration provides rewards for compliant behavior in the short run and attempts to prepare prisoners for release to general population. The differences between the two conditions, however, were not uniformly perceived.

## Discussion

The U.S. prison system houses more than 1.4 million people on any given day (Carson, 2020) and uses restrictive housing settings with some regularity (Beck, 2015). Despite the large-scale use of incarceration, it appears to have reached a state of diminishing returns in terms of reducing criminal and deviant behavior (Liedka et al., 2006). Furthermore, incarceration generally, and restrictive housing specifically, is often criticized for producing adverse effects on prisoner health and psychological well-being (Haney, 2020; Luigi et al., 2020; Massoglia & Pridemore, 2015; Morgan et al., 2016; Porter & Demarco, 2019; Reiter et al., 2020; Strong et al., 2020; Wildeman & Andersen, 2020). A number of initiatives have sought to alleviate the potential harmful effects of incarceration, including a national movement to reform the use of solitary confinement. While much prison reform has occurred via litigation, this study emphasizes how the Oregon DOC has introduced changes proactively through internal reform.

Criminal justice reform is challenging (Jacobs & Olitsky, 2004). It requires motivation to make change and leadership willing to subject itself to criticism internal and external to the system. Instituting new programs also presents thorny challenges in criminal justice settings, especially in prisons. A program as it is described on paper is not always the way it performs in practice (Bourgon & Armstrong, 2005; Gendreau et al., 1999; Rhine et al., 2006). It is also difficult to evaluate the processes and impacts of programs within institutional settings, not least because access is difficult to secure (Duwe & Clark, 2015; Miller & Miller, 2015; Mitchell et al., 2018). All of these issues are especially salient in restrictive housing. Like many areas of criminal justice, the politics of reform far outpace the science, hence the challenges to conduct sound research and evaluation, including randomized controlled trials.

Over the last decade, the Oregon DOC has entered into a period of experimentation and change. The organization invited the Vera Institute of Justice to observe its prison system and make recommendations for reducing their use of restrictive housing. It also sent a delegation of correctional officials to observe and learn from their counterparts in the Norwegian Correctional Service. More recently, the Oregon DOC established a restrictive housing step down reentry program to improve the conditions in these living units, incorporate more rehabilitative programming, and provide incentives for compliant behavior. This study documented the origins, development, and processes of the SUP. The preliminary results of this experiment are tentative but encouraging.

When implementing new policies and practices in prison, it is important to understand the perceptions of correctional officials. Agency leaders, facility managers, line-level officers, and support staff are integral to the prison system and without their buy-in programs and services are unlikely to be implemented as intended (Ahalt et al., 2020; Benefiel, 2019). The interviews with correctional staff in this study revealed a cautious optimism about the SUP, which is a positive sign for the potential success of the SUP. Indeed, had we found otherwise, that custodial staff did not believe in the program, it would not bode well for accomplishing cultural change in restrictive housing, which may be equally if not more important than structural change.

Prisoner accounts of their daily activities suggest that the SUP operates differently than the IMU in several ways. The SUP provides prisoners with more out-of-cell time than offered to those in the IMU. Prisoners have more opportunities to interact with their peers, engage in unit activities (e.g., watching television, participating in yard time), and participate in rehabilitative programming. These changes introduce greater normalcy to restrictive housing custody and, as a consequence, are expected to facilitate smoother reentry to the general prison population and eventually to the community. Although the respondents in the SUP reported communicating with correctional officers slightly less often than those in the IMU, there are two important considerations in interpreting this result. First, prisoners in the SUP may be less reliant on officers for information than those in the IMU because there are more opportunities in the unit to interact with other prisoners. Second, the length of time spent communicating with officers in both groups is decisively brief (2.4 min per day for SUP compared to 4.1 min per day for IMU). While such short durations and the differences reported between SUP and IMU might be an artifact of a prisoners’ reflection on how long they interact with officers in a given week, this finding emphasizes that efforts ought to be made to increase the prosocial interactions between correctional officers and prisoners in both units. Such an endeavor is essential for improving the culture by creating a more humanistic approach in restrictive housing.

Importantly, however, the SUP in Oregon was never fully implemented as intended due to COVID-19. As the impacts of the pandemic begin to wane and prison systems return to more normal operations, the Oregon DOC will be able to increase the provisions offered in the unit (e.g., more out-of-cell time, increased opportunities for socialization with other people, and greater access to programming and other beneficial activities). A stronger dosage of these programmatic elements should serve to further alleviate the potentially harmful aspects of this type of housing which, in turn, could improve indicators of prisoner health and well-being. As we await the opportunity to conduct prospective psychological and behavioral analyses with a larger sample size once the SUP returns to normal operations, this study provides tentative support for the use of step down reentry programs in restrictive housing units.

## Conclusions

The Oregon DOC has undertaken several efforts to reform its use of solitary confinement. As this study highlights, one of those strategies was the development and implementation of a restrictive housing step down reentry program. The authors partnered with the Oregon DOC to conduct a randomized controlled trial of the new SUP. This evaluation revealed encouraging process findings. This research found that it is possible for prison officials to create conditions in restrictive housing environments that may be perceived by prisoners as less harmful and fairer than standard solitary confinement housing. It remains an open empirical question, however, whether step down programs will improve behavior, or if instead they will, through softening punishment, undermine the goals of institutional safety and security. These results, nevertheless, should motivate researchers to investigate the uses and impacts of reentry programs, especially on prisoner populations not included in this study, such as juveniles, women, and individuals with severe mental health diagnoses, recent histories of serious institutional violence and disorder, and who will be released to the community.

## Data Availability

The datasets generated and analyzed during the current study are not publicly available as the project is still in progress but are available from the corresponding author on reasonable request.

## Footnotes

1 On March 12, 2020, the Oregon DOC announced it was suspending all visiting at all 14 state prisons beginning March 13, 2020 (Oregon Department of Corrections, 2021a). The first COVID-19 positive case was reported on April 1, 2020 and was an employee at the Oregon State Penitentiary. On April 2, 2020, the first adult in custody (AIC) within the Oregon DOC tested positive for COVID-19 at Santiam Correctional Institution.

2 Prisoners are eligible for IMU after serving time in disciplinary segregation. The prisoners in our sample spent an average of 131 days (*SD* = 39 days) in disciplinary segregation before being transferred to the IMU.

3 Given the infrequency of prisoners released directly from IMU to the community, our survey sample includes only individuals who were intended to return to the general prison population before their release from prison.

4 Rather than being placed in the IMU, individuals with MH-3 designation are placed in the Behavioral Health Unit, which provides more specialized mental health treatment.

5 Intent-to-treat procedures are standard practice in RCT designs. While RCTs are recognized as the “gold standard” study design, the “incorrect analysis of the data can introduce bias even in the setting of the correct implementation of a valid random allocation sequence” (McCoy, 2017, p. 1075). To preserve the integrity of the randomization process, analysis must be completed according to the group to which participants were originally assigned. Furthermore, estimates from an intent-to-treat analysis are generally conservative (Gupta, 2011).

6 All interviews were transcribed by a professional transcription agency and were stored on a secure server at the University of Colorado Boulder.

7 This collaboration was supported by Amend; a non-profit organization based out of the University of California San Francisco’s School of Medicine that is dedicated to changing the correctional culture in America (Bouffard, 2019).

8 The Oregon DOC operates two IMUs: one for male prisoners at the Snake River Correctional Institution and the other for female prisoners at the Coffee Creek Correctional Facility.

9 Information about the other types of restrictive housing used in Oregon is available in Hastings et al. (2016).

10 An unfortunate impact of the COVID-19 pandemic has been the temporary decreased ability to meet this 5–6 h goal. The prisoners in the SUP, however, still maintained more out-of-cell time than prisoners in the IMU.
